# Complete genome sequence of *Enterococcus faecalis* B05

**DOI:** 10.1128/mra.00395-24

**Published:** 2024-07-08

**Authors:** Jiahao Li, Xiuyun Wu, Jing Meng

**Affiliations:** 1 The State Key Laboratory of Microbial Technology, Shandong University, Qingdao, Shandong, China; 2 Jinan Maternity and Child Care Hospital, Jinan, Shandong, China; Indiana University, Bloomington, Bloomington, Indiana, USA

**Keywords:** genome sequence

## Abstract

*Enterococcus faecalis*, a gram-positive bacterium, is the main intestinal bacteria in humans and animals. We report here the complete genome sequence of *E. faecalis* B05, whose genome length is 2,745,741 bp, containing 2,503 protein-coding genes, with a GC content of 37.64%.

## ANNOUNCEMENT

*Enterococcus faecalis*, a gram-positive, facultative anaerobic bacterium, is one of the main floras in the gut of humans and animals. As intestinal symbiotic bacteria, *Enterococcus* colonizes the digestive system of humans and animals and is involved in the regulation of the immune system (1). *E. faecalis* B05 was isolated in March 2023 from baby feces in Jinan, Shandong Province, China. After 0.1 g sample was added to 1 mL 0.9% sterile sodium chloride solution, the mixture was inoculated into EC (*Escherichia coli)* Broth medium (Purchased from QingDao Hopebio-Technology Co., Ltd.) and cultured at 37°C for 24 h. The enriched bacterial solution was coated on EC agar plates and cultured in an aerobic incubator at 37°C for 24 h. We compared the 16S rRNA genes of single colonies with sequences from the National Center for Biotechnology Information (NCBl) Nucleotide collection database. The colony B05 was identified as *E. faecalis*, and the GenBank accession number of 16S rRNA gene was PP542524.

*E. faecalis* B05 was cultured in EC Broth medium at 37°C for 24 h. Genomic DNA was extracted using the QIAGEN Genomic-tip 20/G kit (QIAGEN). The genome was sequenced using a combination of Illumina Novaseq sequencing platforms and Nanopore PromethION sequencing platforms. For Illumina sequencing, the DNA sample was sheared into 400–500 bp fragments using a Covaris M220 Focused Acoustic Shearer. Illumina sequencing library was prepared from the sheared fragments using the TrueLib DNA Library Rapid Prep Kit (300 cycles). The prepared library then was used for paired-end Illumina sequencing (2 × 150 bp) on an Illumina Novaseq 6,000 platform. For Nanopore sequencing, DNA fragments were recovered using BluePippin’s fully automated nucleic acid recovery system and then attached sequencing adapters supplied in the SQK-LSK109 kit to the DNA ends. The Nanopore library was prepared and sequenced on Oxford Nanopore PromethION48 sequencer with PromethION Flow Cell (R9 Version). All reads were provided as FASTQ files [Illumina, 3,148,922*2 raw pair reads (941,504,344 bases, with 98.46% coverage base on reads mapping and raw Q30 of 91.44%); ONT, 780,000 reads and (807,304,420 bases, with 99.36% coverage based on reads mapping and the largest, average length and reads N50 value of 93,848 bp, 10,350.06 bp, and 17,957 bp)]. The raw data generated by Illumina sequencing was clipped using fastp v0.23.0 to obtain high-quality clean data. Nanopore reads were extracted, basecalled and demultiplexed, and trimmed with the minimum Q score cutoff of 7. Guppy v5.0.16 with high-accuracy mode was used as the base caller (2). The assembly software Unicycler v0.4.8 (3) was used for the ONT genome assembly. Pilon v1.22 software (4) was used to map Illumina short sequences to the assembled genome for correction. Genome annotation was performed using NCBI Prokaryotic Genome Annotation Pipeline (PGAP v5.3) (5). All tools were run with default parameters unless otherwise specified.

*E. faecalis* B05 genome was assembled into four circular replicons, with one circular chromosome (2,730,213 bp) and three circular plasmids (PlasmidA: 7,384 bp; PlasmidB: 5,143 bp; PlasmidC: length, 3,001 bp). The total genome length is 2,745,741 bp, containing 2,503 protein-coding genes, 57 tRNAs, 12 rRNAs, with an average GC content of 37.64%.

## Data Availability

The genome sequence of E. faecalis B05 is available in GenBank under accession numbers to CP139121 –CP139124. The raw sequences are available under Sequence Read Archive (SRA) accession numbers SRX22999955 (Illumina) and SRX22999956 (ONT).
